# A non-pharmacological multidomain intervention of dual-task exercise and social activity affects the cognitive function in community-dwelling older adults with mild to moderate cognitive decline: A randomized controlled trial

**DOI:** 10.3389/fnagi.2023.1005410

**Published:** 2023-03-13

**Authors:** Sangyoon Lee, Kenji Harada, Seongryu Bae, Kazuhiro Harada, Keitaro Makino, Yuya Anan, Takao Suzuki, Hiroyuki Shimada

**Affiliations:** ^1^Department of Preventive Gerontology, Center for Gerontology and Social Science, National Center for Geriatrics and Gerontology, Obu, Japan; ^2^Department of Health Care and Science, Dong-A University, Busan, Republic of Korea; ^3^Graduate School of Human Development and Environment, Kobe University, Kobe, Hyogo, Japan; ^4^Japan Society for the Promotion of Science, Tokyo, Japan; ^5^Kwassui Women’s University, Nagasaki, Japan; ^6^Institute for Aging and Development, J. F. Oberlin University, Machida, Japan

**Keywords:** non-pharmacological multi-domain program, dual task, community-dwelling older adults, cognitive decline, randomized control trial, geriatric health

## Abstract

The present study aimed to determine the impact of a 10-month multidomain program using dual-task exercise and social activity conducted at a community-based facility on improved cognitive function in older adults with mild to moderate cognitive decline. The participants included 280 community-dwelling older adults (age 71–91 years) with mild to moderate cognitive decline. The intervention group exercised for 90 min/day, once a week. Their routine included aerobic exercise and dual-task training which cognitive tasks were performed in combination with exercise. The control group attended health education classes thrice. Before and after the intervention, we measured their cognitive function, physical function, daily conversation, and physical activity. The mean adherence rate of the intervention class was 83.0%. According to a repeated-measures multivariate analysis of covariance in an intent-to-treat analysis, logical memory and 6-min walking distance demonstrated a significant time and group interaction effect. Regarding daily physical activities, we observed significant differences in the daily step count and moderate-to-vigorous physical activity in the intervention group. Our non-pharmacological multidomain intervention resulted in a modest improvement in the cognitive or physical function and building health behavior. It may be a helpful program with a potential role in preventing dementia.

**Clinical Trial Registration**: http://clinicaltrials.gov Identifier ID: UMIN000013097.

## 1. Introduction

In 2015, the population of people aged ≥60 years living with dementia was estimated to be 46.8 million globally; it is expected to continue expansion and reach 131.5 million people in 2050 until the introduction of effective preventative programs (Alzheimer’s Disease International, 2015). Previous studies estimated the prevalence of cognitive impairment without dementia as 22.2% in individuals aged over 71 years in the United States in 2002 (Plassman et al., 2008), whereas the prevalence in Japan with dementia among individuals aged 65 years was 21.1% by 2025 (Cabinet Office, 2017). Despite the difficulty to make an accurate comparison owing to differences in the measured items and the characteristics of the participants, both studies suggested that approximately one-fifth of the elderly population may be experiencing cognitive decline.

Cognitive impairment and dementia are complex multifactorial diseases, and their risk factors vary across lifespans (Livingston et al., 2017). Potentially modifiable lifestyle risk factors include smoking, physical inactivity, diabetes, depression, social isolation, and cognitive inactivity (Livingston et al., 2017). Kivipelto et al. demonstrated that multidomain interventions targeting several risk factors may be an effective strategy to prevent the development of dementia among older adults at a risk (Kivipelto et al., 2018).

Physical exercise programs are a promising non-pharmacological intervention to prevent cognitive decline (van Uffelen et al., 2008; Smith et al., 2010; Livingston et al., 2017). Previous epidemiological studies and meta-analyses on the association between physical activity and cognitive function have reported on greater levels of physical activity–induced improvements in cognitive function among older adults (Snowden et al., 2011; Kirk-Sanchez and McGough, 2013). The World Health Organization (WHO) guidelines on risk reduction for cognitive decline and dementia strongly recommend physical activity for adults. However, recommendations for adults with mild cognitive impairment (MCI) was conditional because of the low quality of evidence (World Health Organization, 2019). The mechanisms by which physical exercise can affect cognitive function are complex and unclear. Physical exercise improves the cerebral blood flow (Ogoh and Ainslie, 2009; Joris et al., 2018), regulates DNA methylation and histone acetylation in the hippocampus, and enhances brain-derived neurotrophic factor (BDNF) expression (Fernandes et al., 2017). Moreover, it reduces amyloid beta load and levels of hyperphosphorylated tau proteins (Lin et al., 2018), increases the cognitive reserve, and prevents memory performance decline (Aguiar et al., 2011; Gomes da Silva et al., 2012).

Multi-task training are effective for morbidity and cognition, compared with a single physical or cognitive exercise. Moreover, dual training is important when considered as a primary prevention and support for healthy aging. Despite studies on the benefits of combining interventions, larger well-designed studies are required; particularly, those regarding the experimental design, sample size, dosage, and outcome selection types (Law et al., 2014; Lauenroth et al., 2016; Zhu et al., 2016). Research based on active control groups is required for older adults with MCI and dementia (Law et al., 2014).

In addition, social participation prevents isolation, which is strongly associated with good health and wellbeing, and should be supported over the life-course according to the WHO guidelines on risk reduction for cognitive decline and dementia (World Health Organization, 2019). However, the guidelines mention about insufficient evidence for supporting the claim (World Health Organization, 2019). There are few randomized controlled trial (RCT) designs that promote social activities because of heterogeneity in measuring such activities. The combined intervention of group-based activities that require social interaction among participants may increase brain stimulation.

Researchers have completed three large RCTs that examined lifestyle interventions to prevent cognitive decline (Ngandu et al., 2015; Moll van Charante et al., 2016; Andrieu et al., 2017); The Finnish Geriatric Intervention Study to Prevent Cognitive Impairment and Disability study examined three large studies that included nutritional guidance, exercise, cognitive training, social activity, and the intensive monitoring of risk factors, and identified targeting interventions to individuals with an elevated risk of dementia as an effective preventive approach (Ngandu et al., 2015). However, methodological issues were apparent from previous studies, such as low adherence, insufficiently intense coaching by trained professionals, and a lack of similarity between the contents of cognitive training and tests used as an outcome (Kivipelto et al., 2018). This necessitates definitive intervention studies to confirm the efficacy of combined programs in preventing cognitive decline.

We aimed to design an RCT to determine the impact of a community-based multidomain intervention using dual-task training and social engagement over 10 months on improved cognitive and physical function and daily activities among community-dwelling older adults.

## 2. Experiment

### 2.1. Design

We performed an RCT of the effects of a multidomain intervention comprising a dual-task exercise and social activity intervention program on community-dwelling older adults with mild to moderate cognitive decline. We specifically analyzed the effects of the program on objective assessments of physical and cognitive function and daily activity as well as the participants’ responses to a questionnaire and accelerometer. The trial (ID: UMIN000013097) is registered at the University Hospital Medical Information Network (UMIN) clinical trials registry website UMIN Clinical Trials Registry,^1^ which is accepted by the International Committee of Medical Journal Editors.

### 2.2. Participants and procedures

The participants were recruited from a sub-cohort of the National Center for Geriatrics and Gerontology-Japan Study of Geriatric Syndrome (NCGG-SGS), which was conducted in 2013 in Midori Ward, Nagoya, Aichi Prefecture, Japan. Nagoya City, located in the central part of Japan, is the capital of the prefecture and an ordinance-designated city. It consists of 16 administrative wards, of which Midori Ward is a suburban residential area, with a total population of 235,631 during the study and 20.2% of them aged over 65 years. This sub-cohort consisted entirely of older adults living in the Midori Ward aged ≥70 years and who were not certified as requiring support or care by the Japanese long-term care insurance system. We recruited 24,271 older adults to participate in a screening survey of physical and cognitive function by mail to individuals, of which 5,257 participated.

Of these 5,257 individuals, 709 were selected as the potential participants after meeting the following inclusion criteria: (1) mild to moderate cognitive decline based on a score ranging from 21 to 24 on the Mini-Mental State Examination (Folstein et al., 1975), (2) no gait dysfunction (walking speed <1 m/s) or other serious health problems at the initial screening, (3) without missing data in the screening items, and (4) not potential participants in other intervention studies. Of the 709 participants, 359 participated in the baseline assessment. As exclusion criteria, 79 participants were eventually excluded because they withdrew their participation (*n* = 24), abnormalities in MRI (*n* = 13), already participated in fitness centers 5 days and over per week (*n* = 7), severe health problem (*n* = 27) and uncompiled data in assessment (*n* = 8).

**Figure 1:**
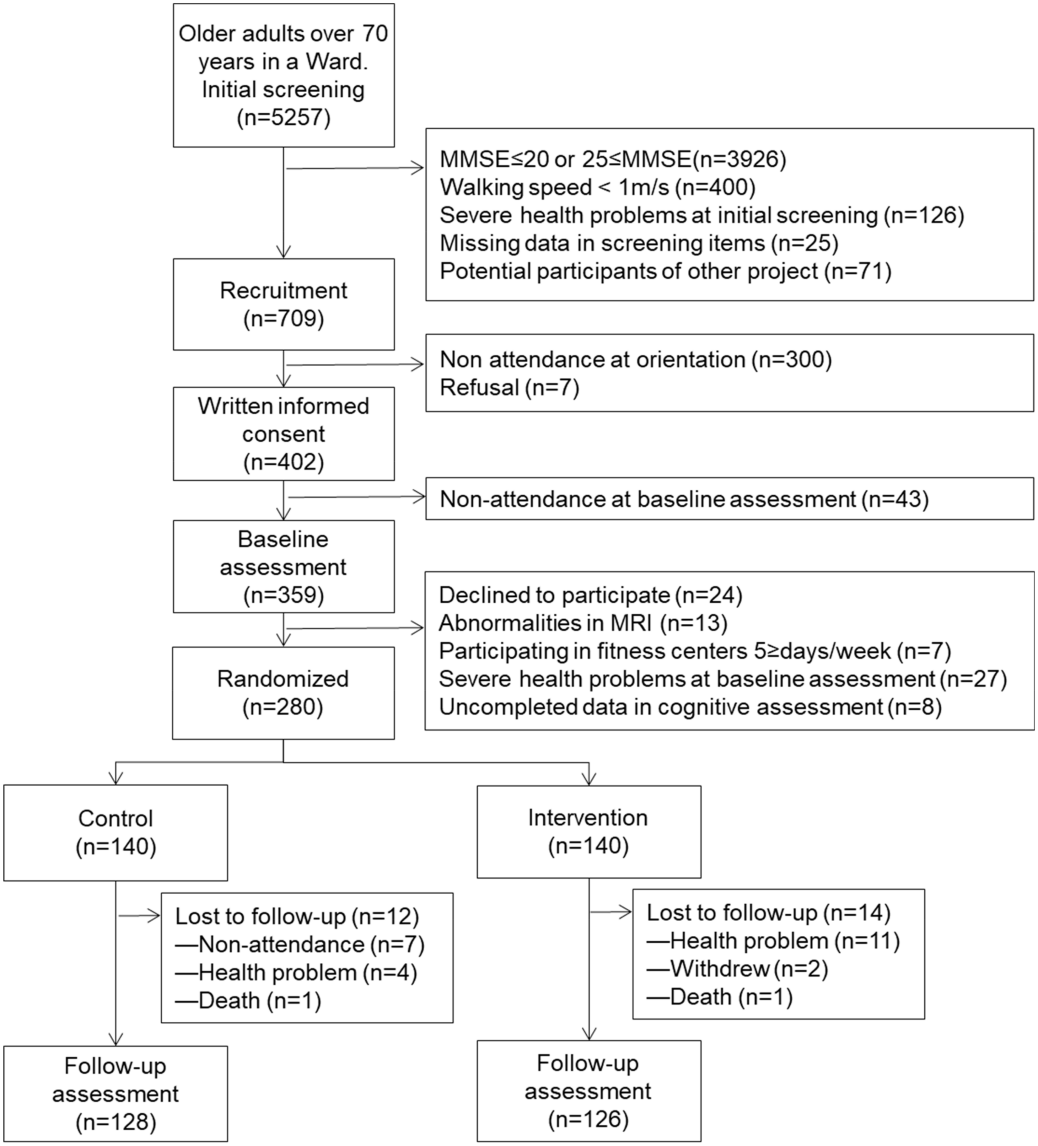
Flow diagram of the participants in the community-based lifestyle intervention program from the screening to the final follow-up assessment. MMSE, mini-mental state examination; MRI, magnetic resonance imaging.

The remaining 280 individuals were allocated to either the intervention or control group (Figure 1). A sample size of 280 was considered sufficient to detect the target effect size with a type 1 error of 5% (*α* = 0.05) and 80% power (*β* = 0.20), according to a power analysis using G*Power 3.1.7 as previous study (Suzuki et al., 2013). We designed the RCT to include two parallel groups, using a 1:1 allocation ratio along with allocation concealment and assessor blinding. The participants were informed of their study group following randomization using computer-generated allocation.

The design of the RCT followed the Consolidated Standards of Reporting Trials. Both the NCGG-SGS and the RCT received prior approval from the Ethics Committee at the National Center for Geriatrics and Gerontology (Approval Number: 637-3). Written informed consent was obtained from all participants in each project.

### 2.3. Interventions

#### 2.3.1. Education program: Control group

Participants randomized into the non-exercise group were offered courses on education three times during the 10 months. They received educational lessons on nutrition and diets, oral care, and healthy longevity for one hour per lesson. The group did not receive specific information regarding exercise, physical activity, or cognitive health related to the intervention program. We provided to all participants the feedback from the test results of older adults who completed the baseline assessment.

#### 2.3.2. Community-based multicomponent program focusing on dual-task exercise and social engagements: Intervention group

Participants in the intervention group joined an exercise course held at local sports facilities once a week that consisted of 19 to 32 participants per group. The exercise course was held once a week for a total of 40 sessions over a 10-month period. They also engaged in mentally stimulating social activities in groups of four or five participants twice per month (14 times in total). One or two trained professionals and trained staff members performed the intervention. The adherence to exercises in the intervention group was 81.4% at follow-up. Participants attended an average of 33 sessions, and the minimum and maximum number of sessions completed ranged from 0 to 40.

Each participant was surveyed about their preferred day of the week, the time of day, and easily accessible fitness facility. Moreover, we determined the fitness facility attended according to their preferences. The exercise classes consisted of two classes per week.

The exercise course lasted approximately 90 min. The exercise course consisted of approximately 15 min of warm-up including stretching, 30 min of aerobic exercise, 30 min of dual-task training, and 15 min of cool-down training. For aerobic exercises, we instructed the participants in stair stepping, brisk walking, or aerobic dance. The mean intensity of the aerobic exercises was targeted at 60% of the maximum heart rate (Lautenschlager et al., 2008). Heart rate was measured by taking the pulse after aerobic exercise (HM06: First Running, Inc.) and perceived exertion was measured using the 10-step Borg rating (RPE). Exercise intensity was calculated as heart rate (HR) reserve. Subjects wore a telemetric HR transmitter on the chest and an HR monitor (RCX-3; Polar Electro, Oy, Finland) on the wrist. The participants solved cognitive tasks while simultaneously performing moderate aerobic exercise to enhance dual-task training (termed “COGNICISE”; Suzuki et al., 2012, 2013; Shimada et al., 2018). In the dual-task training, we requested them to complete mathematical calculations, counting numbers or reciting words forward or backward, executing a word chain, clapping, or following consistent patterns of hand or leg movements along with stair stepping or floor exercises. At the end of the exercise, we instructed the participants in cooling down training with gentle relaxation movements and had a conversation about the content of the next class.

To encourage health behavior changes with reference to the Bandura’s social cognitive model, the participants were given a homework sheet summarizing the exercises they performed each day to enable them to practice at home. Moreover, they performed self-monitoring using monitoring sheets and accelerometers. We instructed them to regularly record their accelerometer step counts and exercise homework on the monitoring sheets. The staff evaluated the sheets and provided positive feedback comments once every 2 weeks.

Mentally stimulating social activities were held twice a month for approximately 1 to 2 h per session, in which the participants engaged in social interactions. Each group member introduced an article or book of their interest and discussed their opinion. The activity contents were recorded in a scrapbook at each session.

### 2.4. Assessments

The assessment was conducted by staff trained in nursing, allied health, or similar qualifications. The assessors were blinded at baseline and at follow-up examinations.

### 2.5. Primary outcomes

#### 2.5.1. Cognitive function

We performed cognitive assessment using the National Center for Geriatrics and Gerontology-Functional Assessment Tool (NCGG-FAT; Makizako et al., 2013b). The NCGG-FAT assesses the word list memory (immediate recognition, delayed recall, and delayed recognition), logical memory (immediate recognition, delayed recall, and delayed recognition), attention and executive function (a tablet version of Trail Making Test (TMT)-part A and B: TMT-A and B), and processing speed (a tablet version of Digit Symbol Substitution Test).

### 2.6. Secondary outcomes

#### 2.6.1. Physical function

We measured the grip strength in kilograms using a Smedley-type handheld dynamometer (GRIP-D; Takei Ltd., Niigata, Japan). The five-times chair stand test (5CS) was used to evaluate the leg strength, and we rapidly recorded the time required to stand and sit five times. The gait speed was measured by the 5-m walking time and expressed in meters per second. We performed the 6-min walking test (6MWT) to estimate the aerobic fitness in older adults (Steffen et al., 2002). The participants were instructed to walk from one end of a 10-m course to the other and return the maximum possible times in 6 min. The distance walked during the 6 min was recorded in meters.

#### 2.6.2. Social engagements

We asked all participants to rate the amount of time they spent talking about health from 0 to 10, where 0 referred to no conversation and 10 referred to the maximum total time of daily conversation regarding health. Their social network was measured using the Lubben Social Network Scale (Lubben, 1988). This scale measures the degree of social engagement including family and friends. The score ranges between 0 and 30, with a higher score indicating more social engagement.

#### 2.6.3. Others

We distributed accelerometers to the participants to measure physical activity such as their daily steps and the time spent performing moderate-to-vigorous physical activity (MVPA) (>three metabolic equivalents) in daily living. Physical activity was measured using an accelerometer with built-in triaxial (GT40-020: Acos Corporation: Nagano, Japan). Participants were instructed to wear the device on their waist on a regular routine basis. Activity intensity levels were measured based on the algorithm of the Kenz Lifecorder (Suzuken Corporation: Nagoya, Aichi, Japan; Kumahara et al., 2004). Activity intensity was estimated on a 10-point scale, with level 4 or higher corresponding to 3 metabolic equivalents or higher. The epoch length of the accelerometer was set up as 4 s, and the daily MVPA time was calculated by summing all the epochs in which the activity intensity was estimated to be level 4 or higher. A valid day for analysis was defined as having ≥10 h of wear time per day for at least 8 days (Gorman et al., 2014). The covariates included the age, gender, education, and medication. To measure other variables regarding clinical characteristics, we used the instrumental activities of daily living (IADL) score (Iwasa et al., 2018), body mass index (BMI), and geriatric depression scale (GDS) score.

### 2.7. Statistical analyses

We compared the baseline characteristics of the groups using a *t*-test and chi-square test for continuous variables and categorical variables, respectively, to determine their homogeneity. We assessed the intervention effects according to the intention-to-treat (ITT) principle with the repeated-measures analysis of covariance (ANCOVA) adjusting for age, gender, educational level and medication at baseline. For the ITT data, we handled missing data using the last observation carried forward method. All statistical analyses were conducted using SPSS Statistics 25 (IBM Corp., Armonk, NY, United States). The significance level α was set at *p* < 0.05.

## 3. Results

### 3.1. Baseline characteristics

The 10-month retention rates were 90.0 and 91.4% for the intervention group and control group, respectively. The main reasons for dropout were health-related problems where 15 participants withdrew and seven participants difficulties in attending. Two participants died during the study. Upon comparing the participants included in the analysis with those lost to follow-up, the included participants displayed higher scores on the cognitive assessments (result not shown).

Table 1 summarizes the characteristics of the participants at baseline. Their mean age was 76.4 (SD = 4.1) years and 39.6% of the participants were women. Moreover, they displayed a mean of 11.9 (SD = 2.6) years of education. The educational level, medication status, IADL, BMI, and GDS score were not significantly different between the groups. Following the allocation, there were no significant group differences in the participant characteristics or their baseline cognitive and physical assessment scores. Tables 2, 3 summarizes the descriptive data and results of the repeated-measures ANCOVA between each within-group analysis of the outcomes. The analyses for per protocol based were limited to 254 participants (90.7%) with cognitive and physical assessment data at both baseline and follow-up (*p* < 0.001). Missing data were imputed for the ITT analysis, such that the additional observations indicated no change from baseline (*n* = 280).

**Table 1:** Demographic and clinical characteristics of the participants at baseline.

Baseline characteristics	Intervention group	Control group	*p* value
	(*n* = 140)	(*n* = 140)	
Age (years), mean (SD)	76.27 (4.06)	76.42 (4.19)	0.761
Women, *n* (%)	59 (53.2)	52 (46.8)	0.464
Education level (years), mean (SD)	11.67 (2.61)	12.11 (2.52)	0.150
Medication (number), mean (SD)	3.03 (2.28)	3.26 (2.93)	0.453
IADL (score), mean (SD)	11.74 (2.78)	11.81 (3.01)	0.837
BMI (score), mean (SD)	22.50 (2.90)	22.82 (2.78)	0.339
GDS (score), mean (SD)	2.76 (2.24)	2.55 (2.25)	0.441

IADL, instrumental activities of daily living; BMI, body mass index; GDS, geriatric depression scale.

**Table 2:** Effects of the intervention and time on the primary outcomes among older adults (Intention-to-treat method and per protocol based).

Measures		Intervention group					Control group					Time × group	
		*n*	Baseline mean (SD)	Post mean (SD)	Change mean (95% CI)	*p*	*n*	Baseline mean (SD)	Post mean (SD)	Change mean (95% CI)	*p*	*F*	*p*
Trail Making Test-part A, time	ITT	140	20.9 (5.2)	21.3 (5.9)	−0.39 (−1.2 to 0.5)		140	20.9 (4.9)	21.0 (4.9)	−0.1 (−0.9 to 0.8)		0.25	
	PPB	125	20.9 (5.2)	21.4 (6.0)	−0.45 (−1.4 to 0.5)		128	20.8 (4.7)	20.9 (4.8)	−0.1 (−1.0 to 0.9)		0.30	
Trail Making Test-part B, time	ITT	140	42.4 (17.0)	42.5 (21.7)	−0.05 (−3.0 to 2.9)		140	43.2 (21.9)	43.2 (18.5)	−0.1 (−3 to 2.9)		0.00	
	PPB	125	41.5 (15.8)	41.5 (21.3)	−0.01 (−3.3 to 3.3)		128	42.9 (22.1)	42.9 (18.3)	−0.1 (−3.4 to 3.2)		0.00	
Digit symbol substitution test, score	ITT	140	52.6 (10.3)	53.2 (10.1)	−0.57 (−1.4 to 0.2)		140	51.6 (9.7)	51.7 (9.8)	−0.1 (−0.9 to 0.7)		0.67	
	PPB	125	53.3 (10.1)	53.9 (9.8)	−0.64 (−1.5 to 0.2)		128	51.8 (9.7)	51.9 (9.7)	−0.1 (−1 to 0.8)		0.68	
Word list memory tasks immediately, score	ITT	140	7.5 (1.3)	7.6 (1.3)	−0.11 (−0.3 to 0.0)		140	7.3 (1.3)	7.5 (1.3)	−0.1 (−0.3 to 0.0)		0.06	
	PPB	125	7.6 (1.1)	7.7 (1.1)	−0.12 (−0.3 to 0.1)		128	7.3 (1.3)	7.4 (1.3)	−0.2 (−0.3 to 0.0)		0.07	
Word list memory tasks, recall, score	ITT	140	3.8 (2.1)	4.0 (2.1)	−0.20 (−0.5 to 0.1)		140	3.6 (2.1)	3.6 (2.1)	0.0 (−0.3 to 0.2)		0.82	
	PPB	125	3.9 (2.1)	4.1 (2.1)	−0.22 (−0.5 to 0.1)		128	3.6 (2.1)	3.6 (2.1)	0.0 (−0.3 to 0.3)		0.77	
Word list memory tasks recognition, score	ITT	140	7.5 (1.5)	7.5 (1.7)	−0.02 (−0.2 to 0.2)		140	7.1 (1.8)	7.2 (1.8)	0.0 (−0.2 to 0.2)		0.01	
	PPB	125	7.6 (1.4)	7.6 (1.5)	−0.03 (−0.3 to 0.2)		128	7.1 (1.9)	7.1 (1.8)	0.0 (−0.3 to 0.2)		0.01	
Logical memory immediately, score	ITT	140	5.4 (2.0)	5.8 (2.1)	−0.37 (−0.7 to −0.1)	*	140	5.4 (1.9)	5.8 (2.2)	−0.4 (−0.7 to −0.2)	**	0.08	
	PPB	125	5.5 (2.0)	5.9 (2.0)	−0.41 (−0.7 to −0.1)	*	128	5.4 (1.9)	5.9 (2.2)	−0.5 (−0.8 to −0.2)	**	0.10	
Logical memory recall, score	ITT	140	5.3 (2.1)	5.7 (2.0)	−0.40 (−0.7 to −0.1)	**	140	5.1 (2.1)	5.6 (2.3)	−0.6 (−0.9 to −0.3)	***	0.78	
	PPB	125	5.4 (2.1)	5.8 (2.0)	−0.44 (−0.7 to −0.1)	**	128	5.1 (2.0)	5.8 (2.2)	−0.6 (−0.9 to −0.3)	***	0.75	
Logical memory recognition, score	ITT	140	6.4 (1.9)	6.9 (1.9)	−0.51 (−0.8 to −0.3)	***	140	6.5 (1.8)	6.6 (2.0)	−0.1 (−0.4 to 0.2)		5.04	*
	PPB	125	6.5 (1.9)	7.1 (1.9)	−0.56 (−0.9 to −0.3)	***	128	6.6 (1.9)	6.7 (2.0)	−0.1 (−0.4 to 0.2)		4.80	*

Adjusted for baseline measurements, age, sex, education, and medication using analyses of covariance. Missing data imputed using last observation carried forward. *p < 0.05, **p < 0.01, and ***p < 0.001. ITT, intention-to-treat; PPB, per protocol based.

**Table 3:** Effects of the intervention and time on the secondary outcomes among older adults (intention-to-treat method and per protocol based).

Measures		Intervention group					Control group					Time × group	
		*n*	Baseline mean (SD)	Post mean (SD)	Change mean (95% CI)	*p*	*n*	Baseline mean (SD)	Post mean (SD)	Change mean (95% CI)	*p*	*F*	*p*
Grip strength, kg	ITT	140	28.2 (7.8)	28.1 (7.5)	0.05 (−0.5 to 0.6)		140	28.4 (7.9)	27.9 (7.8)	0.6 (0.1 to 1.1)	*	1.93	
	PPB	126	28.3 (7.9)	28.2 (7.6)	0.07 (−0.5 to 0.6)		126	28.8 (7.7)	28.2 (7.6)	0.6 (0.1 to 1.2)	*	1.81	
5CS, sec	ITT	140	7.6 (2.2)	7.2 (2.2)	0.39 (0.1 to 0.7)	**	140	7.8 (2.0)	7.6 (2.2)	0.2 (−0.1 to 0.5)		0.62	
	PPB	125	7.6 (2.2)	7.2 (2.3)	0.43 (0.1 to 0.7)	**	126	7.8 (1.9)	7.5 (2.2)	0.3 (−0.1 to 0.6)		0.62	
Walking speed, m/s	ITT	140	1.4 (0.2)	1.4 (0.2)	−0.01 (0.0 to 0.0)		140	1.4 (0.2)	1.3 (0.2)	0.0 (0.0 to 0.3)		1.48	
	PPB	125	1.4 (0.2)	1.4 (0.2)	−0.02 (0.0 to 0.0)		128	1.4 (0.2)	1.3 (0.2)	0.0 (0.0 to 0.0)		1.62	
6MWT, meter	ITT	140	453.5 (60.0)	463.3 (63.6)	−10.05 (−15.1 to −5.0)	***	140	450.0 (54.0)	447.5 (57.4)	2.8 (−2.3 to 7.8)		12.48	***
	PPB	123	456.7 (61.5)	467.8 (64.8)	−11.63 (−17.3 to −5.9)	***	125	452.1 (54.1)	449.3 (57.9)	3.3 (−2.4 to 8.9)		13.25	***
Total time of daily conversation regarding health, score	ITT	137	2.2 (1.9)	3.2 (2.4)	−0.98 (−1.4 to −0.6)	***	140	2.4 (2.1)	2.8 (2.3)	−0.4 (−0.8 to 0.0)		3.74	
	PPB	124	2.2 (1.8)	3.3 (2.4)	−1.08 (−1.5 to −0.6)	***	127	2.4 (2.2)	2.8 (2.3)	−0.4 (−0.9 to 0.0)		3.49	
Social network, score	ITT	140	17.2 (5.2)	17.7 (5.3)	−0.53 (−1.1 to 0.1)		140	17.3 (5.2)	17.0 (5.0)	0.3 (−0.3 to 0.9)		3.80	
	PPB	126	17.4 (5.0)	18.0 (5.1)	−0.60 (−1.2 to 0.0)		128	17.2 (5.1)	16.9 (4.9)	0.3 (−0.3 to 1.0)		4.13	*
Daily steps, step/day	ITT	133	6916.7 (3295.7)	7150.4 (3380.8)	−263.80 (−524.0 to −3.6)	*	134	6478.8 (2589.3)	6243.2 (2633.9)	265.4 (6.2 to 524.6)	*	7.99	**
	PPB	112	6901.7 (3289.5)	7179.2 (3390.1)	−320.14 (−624.8 to −15.5)	*	119	6589.3 (2608.3)	6324.1 (2667.8)	305.3 (9.9 to 600.8)	*	8.34	**
MVPA, min/day	ITT	133	32.9 (23.1)	33.8 (23.3)	−1.14 (−2.9 to 0.6)		134	30.1 (18.6)	28.7 (18.5)	1.6 (−0.2 to 3.4)		4.62	*
	PPB	112	32.5 (23.4)	33.7 (23.6)	−1.39 (−3.5 to 0.7)		119	30.5 (18.9)	28.9 (18.8)	1.8 (−0.2 to 3.8)		4.77	*

Adjusted for baseline measurements, age, sex, education and medication using analyses of covariance. Missing data imputed using last observation carried forward. 5CS, 5-times chair stand test; 6MWT, 6-min walking test; GDS, Geriatric Depression Scale; MVPA, moderate-to-vigorous physical activity; ITT, intention-to-treat; PPB, per protocol based. *p < 0.05, **p < 0.01, and ***p < 0.001.

### 3.2. Primary outcomes

In primary outcomes, we observed significant differences in changes in logical memory for both the intervention and control group between the baseline and post-intervention. At 10 months post-intervention, improvement in immediately logical memory scores were observed in both intervention and control groups (intervention group pre 5.4, post 5.8, *p* < 0.01; control group pre5.4, post 5.8 *p* < 0.05). Recall logical memory scores increased in both intervention and control groups (intervention group pre 5.3, post 5.7, *p* < 0.05; control group pre5.1, post 5.6 *p* < 0.001). Recognition logical memory scores significantly increased in intervention groups (pre 6.4, post 6.9, *p* < 0.001) at 10 month, but not in the control group. The results of recognition logical memory revealed significant interaction by time and group (*F* = 5.04, *p* = 0.026). There were no differences in the mean change from baseline to follow-up between the groups in other cognitive assessments, including TMT-A, TMT-B, and digit symbol substitution, or in secondary assessments between the groups (Table 2).

### 3.3. Secondary outcomes

In secondary outcomes, the results of 6MWT, daily steps and MVPA revealed significant interaction by time and group (6MWT *F* = 12.48, *p* < 0.001; daily steps *F* = 7.99, *p* = 0.005; MVPA *F* = 4.62, *p* = 0.033). Improvement in the results of 5CS, 6MWT, and time spent discussing health were observed in intervention groups (5CS pre 7.6, post 7.2, *p < 0.05*; 6MWT pre 453.5, post 463.3, *p* < 0.001; time spent discussing health pre 2.2, post 3.2, *p* < 0.001). In the control group, the grip strength significantly decreased from baseline post-intervention, but not in the intervention group (pre 28.4, post 27.9, *p* < 0.01). There were no differences in the mean change from baseline to follow-up between the groups in other cognitive assessments, such as walking speed, social network, MVPA. At 10 months post-intervention, the results of daily steps were significantly increased post-intervention in the intervention group from baseline, but control group were decreased from baseline (intervention group pre 6916.7, post 7150.4, *p* < 0.01; control group pre 6478.8, post 6243.2, *p* < 0.01).

### 3.4. Change in outcomes during the 10-month intervention

**Figure 2:**
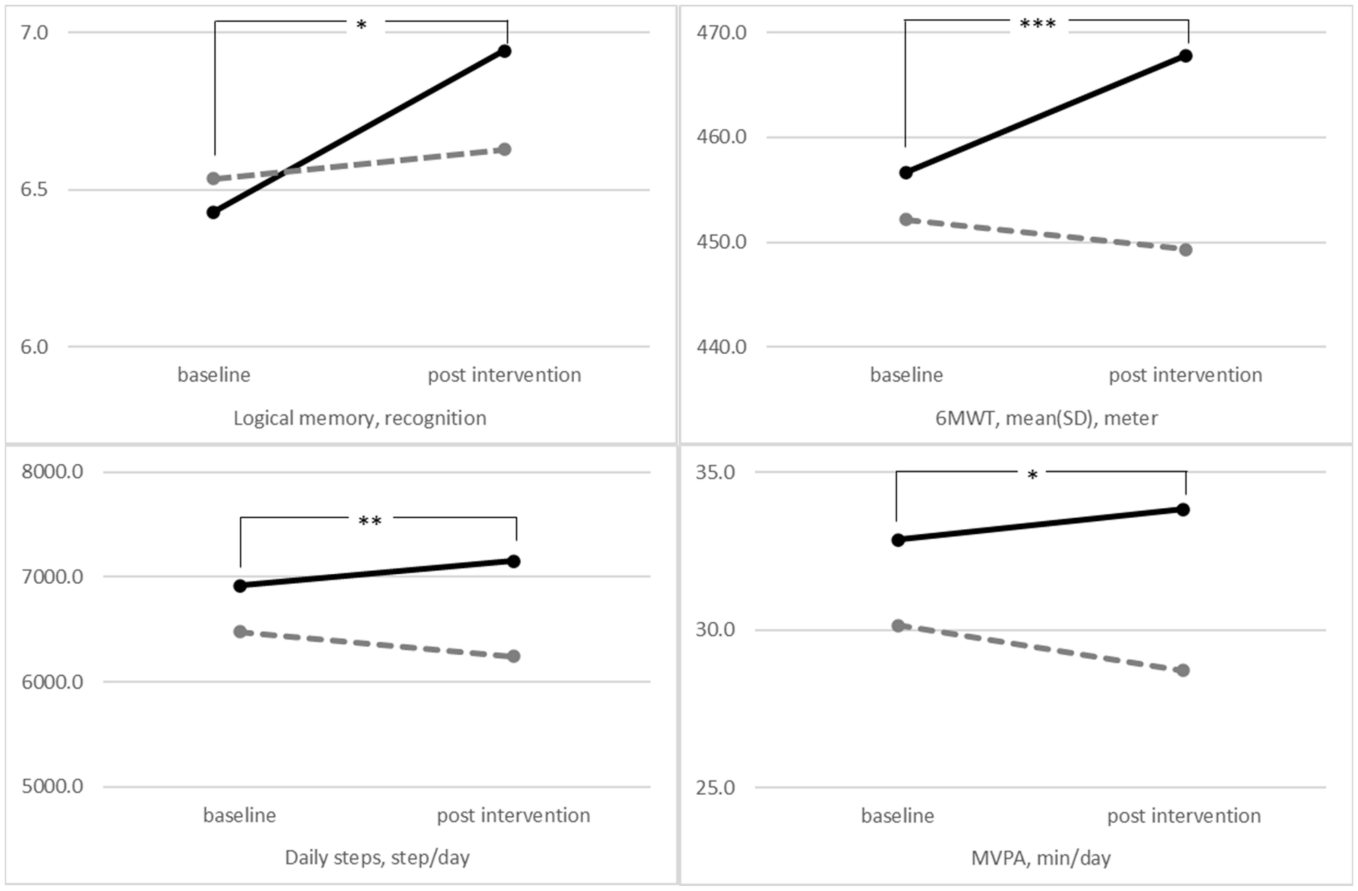
The mean performance of the intervention group (solid lines) and control group (dashed lines) at baseline and 10 months post-intervention after controlling for the covariates values of sex, age, education level, and the number of medications using the repeated-measures analysis of covariance. (Logical memory *p* = 0.026, 6MWT *p* < 0.001, average daily step *p* = 0.005, and MVPA *p* = 0.033). 6MWT, 6-min walking test; MVPA, moderate-to-vigorous physical activity. *p < 0.05, **p < 0.01, and ***p < 0.001.

We noted a significant interaction effect between the time and group for the outcome (Figure 2). We observed significant interaction among recognition logical memory (*p* = 0.026), the distance walked in the 6MWT (*p* < 0.001), average daily steps (*p* = 0.005), and MVPA (*p* = 0.033) in both groups (Figure 2). The improvement in these outcomes at 10 months was better in the intervention group than in the control group, and the results did not change in the per-protocol analysis or in the intention-to-treat analysis (Tables 2, 3).

## 4. Discussion

We aimed to compare the impact of a community-based multicomponent intervention program with a health education program on improved cognitive and physical functions and physical activities in older adults with mild or moderate cognitive decline. Following 10 months, the intervention group displayed significantly greater improvements in logical memory, compared with the control group based on an ITT analysis.

Our study supported previous findings regarding the effects of the combined intervention on memory. Previous studies, including a preliminary clinical trial, have demonstrated that multicomponent interventions using dual tasks exert positive effects on the memory function in older adults (Klusmann et al., 2010; Makizako et al., 2013b; Shimada et al., 2018). A meta-analysis on the combined cognitive and physical exercise intervention demonstrated that combined intervention leads to better memory improvement than either the control group or physical exercise group (Zhu et al., 2016). The findings of this study correspond to these studies (Klusmann et al., 2010; Makizako et al., 2013b; Shimada et al., 2018). Previous reports in combined interventions have suggested a range of 1 to 3 h per week for at least 16 weeks and as long as 6 months (Lauenroth et al., 2016), which would be consistent with the findings of the present study. Unlike previous studies, we did not observe desirable effects on other indices of cognitive functions apart from memory. Lautenschlager et al. (2008) reported that physical activity and behavioral interventions improve the general cognitive function. Another meta-analysis revealed that interventions combining aerobic exercise and strength training improved the working memory, attention, and processing speed (Smith et al., 2010). Large multidomain lifestyle trials on preventing cognitive decline among older adults with an elevated risk of dementia during a 2-year intervention period reported on a beneficial effect on the executive functioning, processing speed, and complex memory tasks (Ngandu et al., 2015). However, there were no differences in the mean change from baseline between the intervention groups at 10 months in the attention (TMT-A), executive functioning (TMT-B), and processing speed (digit symbol substitution). The differences between this study and previous multidomain intervention may be attributed to differences in the participants’ cognitive function, the duration of intervention, and the intensity of the combined cognitive and physical interventions. Moreover, certain cognitive domains, such as memory function, may be particularly sensitive to effects on older adults with cognitive decline, despite short-term interventions.

The intervention group displayed significantly improved 6-min walk distance and daily physical activity, compared with the control group. This distance was used to measure the exercise capacity; the performance of the 6-min walk distance has been associated with better memory function and brain volume among older adults with mild cognitive impairment (Makizako et al., 2013a). Higher levels of step or moderate- and vigorous-intensity activity (Zhu et al., 2015; Calamia et al., 2018) were associated with a lower prevalence of cognitive impairment and better performance. Improvements in daily physical activities indicated that the use of behavior change techniques, such as self-monitoring, receiving positive feedback, and homework, may facilitate the maintenance of walking behavior.

The mechanisms by which interventions comprising dual-task exercise and social activity improve cognition in older people at an increased risk of dementia are unclear. Physical activity is associated with amyloid clearance or cognitive reserve (Fratiglioni et al., 2004; Kivipelto et al., 2018); increasing brain volume (Rovio et al., 2010) as well as BDNF levels may play a role in this association (Komulainen et al., 2008). Furthermore, a healthy lifestyle may decrease the rate of cognitive decline with aging and delay the onset of cognitive symptoms in age-associated diseases (Murman, 2015). The intervention program may not only improve these healthy lifestyle factors, including physical, cognitive, and social engagement, but also exert an intermediate effect by expanding the social network (Seeman and Crimmins, 2001), treating depression, improving self-esteem, and managing stress (Fratiglioni et al., 2004).

Our findings suggest that lifestyle interventions, including the dual-task exercise of cognitive and physical training and social activity, improved the cognitive and physical function in community-dwelling older adults with cognitive decline. However, we should address several limitations of these results. First, the multicomponent intervention, including moderate physical activity, was sufficient to partially increase the cognitive function; however, it may have been insufficient to produce other cognitive effects. Second, we followed participants for 10 months, which may have been a relatively short period to affect the incidence of cognitive decline-related dementia in the long term. Currently, we are following dementia onset in this group of participants. We intend to report on the effects on dementia onset in the future. Third, we did not account for the mechanistic genetic outcomes, such as apolipoprotein E ε4 genotype. There were no other significant differences in the baseline characteristics in terms of cognitive performance. Further studies are needed to determine the impact of the multicomponent intervention trials on the onset of dementia.

## 5. Conclusion

Our non-pharmacological multidomain intervention demonstrated a modest improvement in the cognitive or physical function and building health behavior in older adults with mild-to-moderate cognitive decline. Over 46 million people will have dementia by 2050, thus necessitating preventative interventions to delay or halt its progression. This program may be beneficial for older adults with mild-to-moderate cognitive decline in preventing the development of dementia.

## Data availability statement

The datasets presented in this article are not readily available due to ethical restrictions. Requests to access the datasets should be directed to SL, sylee@ncgg.go.jp.

## Ethics statement

The studies involving human participants were reviewed and approved by Committee at the National Center for Geriatrics and Gerontology (Approval Number: 637-3). The patients/participants provided their written informed consent to participate in this study.

## Author contributions

SL: conceptualization, methodology, and writing—original draft preparation. SL and KaH: methodology. SL, KeH, SB, KaH, KM, and YA: investigation. SL, KeH, SB, KaH, KM, YA, TS, and HS: writing—review and editing. TS: supervision. HS: project administration and funding acquisition. All authors have read and agreed to the published version of the manuscript.

## Funding

This work was funded by Strategic Basic Research Programs Redesigning Communities for Aged Society (RISTEX) of the Japan Science and Technology Agency (JST), Health and Labor Sciences Research Grants from the Japanese Ministry of Health, Labour and Welfare (H24-tyoujyu-ippan-004, the Research Funding for Longevity Sciences (25-26, 21-45) from the National Center for Geriatrics and Gerontology, Grant-in-Aid for Scientific Research C (No. 20K11276), and joint research with Kao Corporation.

## Conflict of interest

The authors declare that the research was conducted in the absence of any commercial or financial relationships that could be construed as a potential conflict of interest.

## Publisher’s note

All claims expressed in this article are solely those of the authors and do not necessarily represent those of their affiliated organizations, or those of the publisher, the editors and the reviewers. Any product that may be evaluated in this article, or claim that may be made by its manufacturer, is not guaranteed or endorsed by the publisher.

## Footnotes

^1^ http://www.umin.ac.jp/ctr/index.htm

## References

[ref1] Aguiar A. S. Jr. Castro A. A. Moreira E. L. Glaser V. Santos A. R. S. Tasca C. I.. (2011). Short bouts of mild-intensity physical exercise improve spatial learning and memory in aging rats: involvement of hippocampal plasticity via AKT, CREB, and BDNF signaling. Mech. Ageing Dev. 132, 560–567. doi: 10.1016/j.mad.2011.09.005 21983475

[ref2] Alzheimer’s Disease International. (2015).World Alzheimer report 2015. Available at: https://www.alz.co.uk/research/WorldAlzheimerReport2015.pdf (Accessed June 23, 2022).

[ref3] Andrieu S. Guyonnet S. Coley N. Cantet C. Bonnefoy M. Bordes S.. (2017). Effect of long-term omega 3 polyunsaturated fatty acid supplementation with or without multidomain intervention on cognitive function in elderly adults with memory complaints (MAPT): a randomised, placebo-controlled trial. Lancet Neurol. 16, 377–389. doi: 10.1016/S1474-4422(17)30040-6 28359749

[ref4] Cabinet Office (2017). Heisei 29th edition of the white book on ageing society (outline edition). [Online; in Japanese]. Available at: https://www8.cao.go.jp/kourei/whitepaper/w-2017/html/gaiyou/s1_2_3.html. (Accessed February 23, 2021).

[ref5] Calamia M. De Vito A. Bernstein J. P. K. Weitzner D. S. Carmichael O. T. Keller J. N. (2018). Pedometer-assessed steps per day as a predictor of cognitive performance in older adults. Neuropsychology 32, 941–949. doi: 10.1037/neu0000487 30080077

[ref6] Fernandes J. Arida R. M. Gomez-Pinilla F. (2017). Physical exercise as an epigenetic modulator of brain plasticity and cognition. Neurosci. Biobehav. Rev. 80, 443–456. doi: 10.1016/j.neubiorev.2017.06.012 28666827 PMC5705447

[ref7] Folstein M. F. Folstein S. E. McHugh P. R. (1975). "mini-mental state". A practical method for grading the cognitive state of patients for the clinician. J. Psychiatr. Res. 12, 189–198. doi: 10.1016/0022-3956(75)90026-6 1202204

[ref8] Fratiglioni L. Paillard-Borg S. Winblad B. (2004). An active and socially integrated lifestyle in late life might protect against dementia. Lancet Neurol. 3, 343–353. doi: 10.1016/S1474-4422(04)00767-7 15157849

[ref9] Gomes da Silva S. Unsain N. Mascó D. H. Toscano-Silva M. de Amorim H. A. Araújo B. H.. (2012). Early exercise promotes positive hippocampal plasticity and improves spatial memory in the adult life of rats. Hippocampus 22, 347–358. doi: 10.1002/hipo.20903 21136521

[ref10] Gorman E. Hanson H. M. Yang P. H. Khan K. M. Liu-Ambrose T. Ashe M. C. (2014). Accelerometry analysis of physical activity and sedentary behavior in older adults: a systematic review and data analysis. Eur. Rev. Aging Phys. Act. 11, 35–49. doi: 10.1007/s11556-013-0132-x 24765212 PMC3990855

[ref11] Iwasa H. Masui Y. Inagaki H. Yoshida Y. Shimada H. Otsuka R.. (2018). Assessing competence at a higher level among older adults: development of the Japan Science and Technology Agency index of competence (JST-IC). Aging Clin. Exp. Res. 30, 383–393. doi: 10.1007/s40520-017-0786-8 28646250

[ref12] Joris P. J. Mensink R. P. Adam T. C. Liu T. T. (2018). Cerebral blood flow measurements in adults: a review on the effects of dietary factors and exercise. Nutrients 10:530. doi: 10.3390/nu10050530, PMID: 29693564 PMC5986410

[ref13] Kirk-Sanchez N. J. McGough E. L. (2013). Physical exercise and cognitive performance in the elderly: current perspectives. Clin. Interv. Aging 9, 51–62. doi: 10.2147/CIA.S39506 24379659 PMC3872007

[ref14] Kivipelto M. Mangialasche F. Ngandu T. (2018). Lifestyle interventions to prevent cognitive impairment, dementia and Alzheimer disease. Nat. Rev. Neurol. 14, 653–666. doi: 10.1038/s41582-018-0070-3 30291317

[ref15] Klusmann V. Evers A. Schwarzer R. Schlattmann P. Reischies F. M. Heuser I.. (2010). Complex mental and physical activity in older women and cognitive performance: a 6-month randomized controlled trial. J. Gerontol. A Biol. Sci. Med. Sci. 65A, 680–688. doi: 10.1093/gerona/glq053 20418350

[ref16] Komulainen P. Pedersen M. Hänninen T. Bruunsgaard H. Lakka T. A. Kivipelto M.. (2008). BDNF is a novel marker of cognitive function in ageing women: the DR's EXTRA study. Neurobiol. Learn. Mem. 90, 596–603. doi: 10.1016/j.nlm.2008.07.014 18707012

[ref17] Kumahara H. Schutz Y. Ayabe M. Yoshioka M. Yoshitake Y. Shindo M.. (2004). The use of uniaxial accelerometry for the assessment of physical-activity-related energy expenditure: a validation study against whole-body indirect calorimetry. Br. J. Nutr. 91, 235–243. doi: 10.1079/BJN20031033 14756909

[ref18] Lauenroth A. Ioannidis A. E. Teichmann B. (2016). Influence of combined physical and cognitive training on cognition: a systematic review. BMC Geriatr. 16, 1–14. doi: 10.1186/s12877-016-0315-1 27431673 PMC4950255

[ref19] Lautenschlager N. T. Cox K. L. Flicker L. Foster J. K. van Bockxmeer F. M. Xiao J.. (2008). Effect of physical activity on cognitive function in older adults at risk for Alzheimer disease: a randomized trial. JAMA 300, 1027–1037. doi: 10.1001/jama.300.9.1027 18768414

[ref20] Law L. L. Barnett F. Yau M. K. Gray M. A. (2014). Effects of combined cognitive and exercise interventions on cognition in older adults with and without cognitive impairment: a systematic review. Ageing Res. Rev. 15, 61–75. doi: 10.1016/j.arr.2014.02.008 24632497

[ref21] Lin T. W. Tsai S. F. Kuo Y. M. (2018). Physical exercise enhances neuroplasticity and delays Alzheimer's disease. Brain Plast. 4, 95–110. doi: 10.3233/BPL-180073 30564549 PMC6296269

[ref22] Livingston G. Sommerlad A. Orgeta V. Costafreda S. G. Huntley J. Ames D.. (2017). Dementia prevention, intervention, and care. Lancet 390, 2673–2734. doi: 10.1016/S0140-6736(17)31363-6 28735855

[ref23] Lubben J. E. (1988). Assessing social networks among elderly populations. Fam. Community Health 11, 42–52. doi: 10.1097/00003727-198811000-00008

[ref24] Makizako H. Shimada H. Doi T. Park H. Yoshida D. Suzuki T. (2013b). Six-minute walking distance correlated with memory and brain volume in older adults with mild cognitive impairment: a voxel-based morphometry study. Dement. Geriatr. Cogn. Dis. Extra 3, 223–232. doi: 10.1159/000354189, PMID: 24052797 PMC3776400

[ref25] Makizako H. Shimada H. Park H. Doi T. Yoshida D. Uemura K.. (2013a). Evaluation of multidimensional neurocognitive function using a tablet personal computer: test-retest reliability and validity in community-dwelling older adults. Geriatr Gerontol Int 13, 860–866. doi: 10.1111/ggi.12014 23230988

[ref26] Moll van Charante E. P. Richard E. Eurelings L. S. van Dalen J. W. Ligthart S. A. van Bussel E. F.. (2016). Effectiveness of a 6-year multidomain vascular care intervention to prevent dementia (preDIVA): a cluster-randomised controlled trial. Lancet 388, 797–805. doi: 10.1016/S0140-6736(16)30950-3 27474376

[ref27] Murman D. L. (2015). The impact of age on cognition. Semin. Hear. 36, 111–121. doi: 10.1055/s-0035-1555115 27516712 PMC4906299

[ref28] Ngandu T. Lehtisalo J. Solomon A. Levälahti E. Ahtiluoto S. Antikainen R.. (2015). A 2 year multidomain intervention of diet, exercise, cognitive training, and vascular risk monitoring versus control to prevent cognitive decline in at-risk elderly people (FINGER): a randomised controlled trial. Lancet 385, 2255–2263. doi: 10.1016/S0140-6736(15)60461-5 25771249

[ref29] Ogoh S. Ainslie P. N. (2009). Cerebral blood flow during exercise: mechanisms of regulation. J. Appl. Physiol. 107, 1370–1380. doi: 10.1152/japplphysiol.00573.2009 19729591

[ref30] Plassman B. L. Langa K. M. Fisher G. G. Heeringa S. G. Weir D. R. Ofstedal M. B.. (2008). Prevalence of cognitive impairment without dementia in the United States. Ann. Intern. Med. 148, 427–434. doi: 10.7326/0003-4819-148-6-200803180-00005 18347351 PMC2670458

[ref31] Rovio S. Spulber G. Nieminen L. J. Niskanen E. Winblad B. Tuomilehto J.. (2010). The effect of midlife physical activity on structural brain changes in the elderly. Neurobiol. Aging 31, 1927–1936. doi: 10.1016/j.neurobiolaging.2008.10.007 19062136

[ref32] Seeman T. E. Crimmins E. (2001). Social environment effects on health and aging: integrating epidemiologic and demographic approaches and perspectives. Ann. N. Y. Acad. Sci. 954, 88–117. doi: 10.1111/j.1749-6632.2001.tb02749.x 11797869

[ref33] Shimada H. Makizako H. Doi T. Park H. Tsutsumimoto K. Verghese J.. (2018). Effects of combined physical and cognitive exercises on cognition and mobility in patients with mild cognitive impairment: a randomized clinical trial. J. Am. Med. Dir. Assoc. 19, 584–591. doi: 10.1016/j.jamda.2017.09.019 29153754

[ref34] Smith P. J. Blumenthal J. A. Hoffman B. M. Cooper H. Strauman T. A. Welsh-Bohmer K.. (2010). Aerobic exercise and neurocognitive performance: a meta-analytic review of randomized controlled trials. Psychosom. Med. 72, 239–252. doi: 10.1097/PSY.0b013e3181d14633 20223924 PMC2897704

[ref35] Snowden M. Steinman L. Mochan K. Grodstein F. Prohaska T. R. Thurman D. J.. (2011). Effect of exercise on cognitive performance in community-dwelling older adults: review of intervention trials and recommendations for public health practice and research. J. Am. Geriatr. Soc. 59, 704–716. doi: 10.1111/j.1532-5415.2011.03323.x 21438861

[ref36] Steffen T. M. Hacker T. A. Mollinger L. (2002). Age- and gender-related test performance in community-dwelling elderly people: six-minute walk test, berg balance scale, timed up & go test, and gait speeds. Phys. Ther. 82, 128–137. doi: 10.1093/ptj/82.2.128 11856064

[ref37] Suzuki T. Shimada H. Makizako H. Doi T. Yoshida D. Ito K.. (2013). A randomized controlled trial of multicomponent exercise in older adults with mild cognitive impairment. PLoS One 8:e61483. doi: 10.1371/journal.pone.0061483, PMID: 23585901 PMC3621765

[ref38] Suzuki T. Shimada H. Makizako H. Doi T. Yoshida D. Tsutsumimoto K.. (2012). Effects of multicomponent exercise on cognitive function in older adults with amnestic mild cognitive impairment: a randomized controlled trial. BMC Neurol. 12:12. doi: 10.1186/1471-2377-12-128, PMID: 23113898 PMC3534485

[ref39] Van Uffelen J. G. Chin A. P. M. J. Hopman-Rock M. van Mechelen W. (2008). The effects of exercise on cognition in older adults with and without cognitive decline: a systematic review. Clin. J. Sport Med. 18, 486–500. doi: 10.1097/JSM.0b013e3181845f0b 19001882

[ref40] World Health Organization. (2019). World Health Organization. Available at: https://apps.who.int/iris/bitstream/handle/10665/312180/9789241550543-eng.pdf?ua=1

[ref41] Zhu W. Howard V. J. Wadley V. G. Hutto B. Blair S. N. Vena J. E.. (2015). Association between objectively measured physical activity and cognitive function in older adults-the reasons for geographic and racial differences in stroke study. J. Am. Geriatr. Soc. 63, 2447–2454. doi: 10.1111/jgs.13829 26691697 PMC4688903

[ref42] Zhu X. Yin S. Lang M. He R. Li J. (2016). The more the better? A meta-analysis on effects of combined cognitive and physical intervention on cognition in healthy older adults. Ageing Res. Rev. 31, 67–79. doi: 10.1016/j.arr.2016.07.003 27423932

